# Novel viral vectors in infectious diseases

**DOI:** 10.1111/imm.12829

**Published:** 2017-09-26

**Authors:** Ian R. Humphreys, Sarah Sebastian

**Affiliations:** ^1^ Institute of Infection and Immunity/Systems Immunity University Research Institute Cardiff University Cardiff UK; ^2^ The Wellcome Trust Sanger Institute Hinxton UK; ^3^ The Jenner Institute University of Oxford Oxford UK

**Keywords:** memory, T cell, vaccination, viral

## Abstract

Since the development of vaccinia virus as a vaccine vector in 1984, the utility of numerous viruses in vaccination strategies has been explored. In recent years, key improvements to existing vectors such as those based on adenovirus have led to significant improvements in immunogenicity and efficacy. Furthermore, exciting new vectors that exploit viruses such as cytomegalovirus (CMV) and vesicular stomatitis virus (VSV) have emerged. Herein, we summarize these recent developments in viral vector technologies, focusing on novel vectors based on CMV, VSV, measles and modified adenovirus. We discuss the potential utility of these exciting approaches in eliciting protection against infectious diseases.

## Introduction

Recombinant viral vectors represent promising vaccine platforms due to their ability to express heterologous antigens and induce antigen‐specific cellular immune responses in addition to robust antibody titres, without the need for exogenous adjuvants. Vaccinia virus was the first virus to be developed as a vaccine vector,^1^ and numerous others have since been explored as delivery vehicles for foreign immunogens. Here, we discuss a selection of novel vaccine vectors (Table 1) that have entered clinical trials recently and/or are forerunners for licensure. As a number of other viral vectors have already been comprehensively reviewed,^2^, ^3^, ^4^, ^5^, ^6^, ^7^ we will not discuss them further.

**Table 1:** Viral vaccine vectors discussed in this review and their characteristics

Vector	Type of virus	Cargo capacity (kb)	Predominant immune response	Clinical development stage
Cytomegalovirus	DNA	>6	CD4^+^, CD8^+^ and antibodies	Phase I
Novel adenoviruses	DNA	7	CD8^+^ and antibodies	Phase II
Vesicular stomatitis virus	‐ssRNA	6	Antibodies and some CD4^+^, CD8^+^	Phase III
Measles virus	‐ssRNA	>6	CD4^+^ and antibodies	Phase I

## Cytomegalovirus

Human cytomegalovirus (HCMV) is a β‐herpesvirus with a large (236 kbp) DNA genome that establishes life‐long, usually asymptomatic, infection in healthy individuals. Significant interest in harnessing HCMV in vaccine vector development has stemmed from the observation that HCMV induces unusually large T‐cell responses (reviewed elsewhere^8^). Natural infection with HCMV elicits broad T‐cell responses directed to a vast array of antigens, with HCMV‐specific responses comprising ~10% of the entire CD4^+^ and CD8^+^ T‐cell memory compartments.^9^ Although HCMV employs numerous immune evasion strategies to avoid control by the host immune system (reviewed in refs 10, 11), HCMV‐specific T‐cell responses are long‐lived, with particularly high frequencies in elderly individuals.^12^ Human CMV‐specific T cells also maintain functionality during virus chronicity and readily produce multiple effector molecules (e.g. interferon‐*γ*, tumour necrosis factor‐*α*) upon stimulation^13^, ^14^ and control virus replication *in vitro*.^15^ Furthermore, experiments in the murine CMV (mCMV) model that recapitulates the accumulation of highly functional CMV‐specific T cells over time^16^, ^17^, ^18^ demonstrate that CMV infection triggers seeding of tissue‐resident memory T cells in peripheral, including mucosal, tissues.^19^, ^20^ Hence, although HCMV also induces substantial antibody responses upon infection,^21^ this virus represents a particularly exciting tool for inducing potent T‐cell immunity.

Human CMV exhibits several other properties attractive for viral vectors. CMV‐based vectors can be engineered to express multiple exogenous immunogens.^22^, ^23^ Human CMV also super‐infects HCMV‐immune hosts.^24^ The immunogenicity of CMV‐based vaccines was first investigated using recombinant mCMV‐based constructs. These induced accumulation of T cells reactive to peptides derived from heterologous antigens that, importantly, conferred protection from heterologous (vaccinia) viral challenge.^25^ Subsequently, induction of protective pathogen‐specific T‐cell responses by mCMV‐based vectors has been demonstrated in Ebola virus, herpes simplex virus and *Mycobacterium tuberculosis* challenge models,^26^, ^27^, ^28^, ^29^ although non‐specific induction of natural killer cell responses partially contributes to early anti‐mycobacterial activity of mCMV.^28^

Broad interest in HCMV‐based vaccines was triggered by encouraging data from rhesus CMV (RhCMV) ‐based vaccines engineered to express antigens from simian immunodeficiency virus (SIV). Using vectors expressing SIV Gag, Env and a Rev‐Tat‐Nef fusion protein, Picker and colleagues demonstrated vaccine‐induced protection from mucosal SIV challenge that was associated with the development of effector memory T‐cell responses.^22^, ^23^ Whereas RhCMV‐induced protection did not preclude SIV spread from mucosal sites of entry, progressive clearance of SIV in ~ 50% macaques was reported, providing evidence that CMV‐based vaccines may be exploited to induce protective anti‐HIV immunity.^30^, ^31^

Analysis of RhCMV vaccine‐induced T‐cell immunity revealed broad CD8^+^ T‐cell responses with an altered epitope hierarchy to responses induced by natural SIV infection. Intriguingly, RhCMV‐induced CD8^+^ T cells were restricted by MHC class II^30^ and HLA‐E^32^ rather than through classical MHC‐Ia restriction. This unique induction of CD8^+^ T‐cell responses was attributed to deletion of the Rh157.5/4 genes within the fibroblast‐adapted RhCMV vector.^32^ Rh157.5/4 are orthologues of the HCMV UL128/UL130 genes that encode components of the viral pentameric complex that is required for viral entry into non‐fibroblast cells.^33^ How deletion of these genes leads to the induction of unique T‐cell responses is unclear. Interestingly, in a phase I trial in humans using chimeric HCMV that lacked the pentameric complex, vaccine‐induced CD8^+^ T‐cell responses exhibited classical MHC restriction.^34^ This may indicate key biological differences between RhCMV and HCMV, or may reflect the incomplete understanding of how RhCMV vectors elicit their unusual responses. It will be important to define the mechanisms that underpin the induction of these unusual T‐cell responses, and to identify which responses are critical for protection, to generate human CMV vectors that are equally effective.

Cytomegalovirus‐based vectors are clearly exciting. However, HCMV is pathogenic, and attenuated vectors are necessary for translation into humans. In mice, temperature‐sensitive mCMV fails to induce robust virus‐specific CD8^+^ T‐cell memory,^35^ eliminating this strategy from exploration for vector attenuation. Encouragingly, however, spread‐deficient mCMV vectors lacking the surface glycoprotein L^36^ or the virion protein M94^37^ induce robust T‐cell immunity. Indeed, glycoprotein L‐deficient (ΔgL) mCMV induces circulating effector memory‐like CD8^+^ T‐cell responses, although the degree to which different mCMV‐specific responses are induced varies substantially.^36^ Whether variation reflects the differential dependence of mCMV‐specific CD8^+^ T cells on CD4^+^ T‐cell help^38^, ^39^, ^40^ is unclear. Importantly, we observed that gL deficiency substantially impairs the seeding of multiple epitope‐specific CD8^+^ T‐cell responses within peripheral tissues, and ΔgL mCMV‐induced CD8^+^ T cells exhibit sub‐optimal recall responsiveness (I.R. Humphreys, unpublished observation). Hence, a greater understanding of how to safely induce potent T‐cell responses using CMV‐based vectors will inform future strategies.

Studying CMV‐induced T‐cell immunity may also inform alternative vector‐based vaccine strategies. Experiments with mCMV have suggested that antigen expression rather than peptide‐intrinsic properties influence mCMV‐induced T‐cell expansions.^29^ Furthermore, C‐terminal localization of peptide in viral proteins greatly increases peptide availability for proteosomal processing and subsequent accumulation of protective peptide‐specific T‐cell memory.^41^ Interestingly, adenovirus‐based vectors engineered to express peptide mini‐genes can induce effector memory T‐cell accumulations indicative of CMV‐induced T‐cell immunity.^42^, ^43^ Hence, studies of CMV‐induced T‐cell responses may inform the development of alternative viral vector systems capable of inducing robust effector memory T‐cell responses.

## Enhanced adenoviral vectors

Soon after their pioneering development as gene therapy vectors in the early 1990s, adenoviruses were also explored as vaccine vectors^44^ and have been used in numerous clinical vaccine trials since 2003.^45^ Hence, they are not considered novel vectors *per se*. However, in the past few years, several groups have made innovative improvements to adenoviral vectors that are worth exploring here because they are already, or have the potential to become, clinically relevant. The various enhancements broadly address two challenges: (i) overcoming pre‐existing anti‐vector immunity and (ii) enhancing vaccine‐induced antigen‐specific immunogenicity.

Adenoviruses are non‐enveloped icosahedral viruses with 30–40 kb linear DNA genomes, which can be genetically manipulated without difficulty. The antigen expression cassette is most often inserted into the E1 genomic locus, rendering the virus replication‐deficient. Vectors in which non‐essential E3 genes are also deleted can accommodate expression cassettes up to a size of 7 kb. Until recently, replication‐deficient AdHu5 was the most widely used adenovirus vector in vaccine development, because of its ability to elicit exceptionally strong CD8^+^ T‐cell and antibody responses, and the ability to generate high titres of virus during manufacturing. However, pre‐existing immunity against this vector, specifically neutralizing antibody titre, was shown to correlate with a reduction in antigen‐specific immunogenicity in clinical trials.^46^, ^47^ Many groups consequently explored adenoviruses with lower seroprevalence in the human population, such as different human adenovirus serotypes or simian adenoviruses. Interestingly, different serotypes were found to elicit different immunogenicity profiles in mice with respect to phenotype, function and longevity of the cellular immune response.^48^ For example, the human adenoviral vectors Ad26 and Ad35 induced enhanced memory CD8^+^ T cells and more polyfunctional CD8^+^ T cells compared with Ad5.^48^ These two alternative vectors (Ad26 and Ad35) have also been evaluated in clinical trials, with variable outcomes.^49^, ^50^, ^51^, ^52^ For example, Ad26 and Ad35 vectors containing the HIV‐1 env antigen were used in heterologous prime‐boost combinations in a Phase I trial.^53^ The authors found that the Ad26‐Ad35 prime‐boost elicited significantly higher antibody titres than the Ad35‐Ad26 regimen, but T‐cell responses were modest overall. Ad26 was also used as a priming vector in Ebola clinical trials, where together with a Modified Vaccinia virus Ankara (MVA) boost, vaccination was able to elicit a strong and durable antibody response to the Ebola virus antigen.^54^, ^55^ Ad35 has additionally been evaluated in several other HIV vaccine trials,^51^, ^56^, ^57^, ^58^ where it was demonstrated to be safe and immunogenic. Furthermore, in a tuberculosis vaccine trial it was found to be safe in both infants and HIV^+/−^ adults. However, it only elicited a cellular immune response upon repeated high‐dose vaccinations.^49^

In addition to human adenoviruses, many simian adenovirus‐based vaccine vectors have been tested preclinically, and five have advanced to clinical studies to date,^59^, ^60^, ^61^ with promising results. A prominent example is ChAd3‐EBOZ, a chimpanzee adenoviral vector encoding the Ebola Zaire glycoprotein, which was evaluated in Phase I and II clinical trials in response to the recent Ebola epidemic.^62^, ^63^, ^64^ This vector was assessed with and without an MVA booster dose, and was found to elicit strong antibody and T‐cell responses, which could be increased in magnitude and durability by an MVA boost. Another chimpanzee adenoviral vector, ChAd63, has also been evaluated in several clinical trials (malaria,^65^, ^66^, ^67^ leishmaniasis^68^) with results showing excellent safety and immunogenicity, even in infants and children.

Improving transgene immunogenicity is also a significant focus of ongoing studies. One strategy to enhance the immune response against exogenous antigen is to increase immunogen production from the vaccine vector. This is difficult to achieve with replication‐deficient (E1‐deleted) adenovirus vectors, as antigen expression is restricted to the single copy of the vector genome present in the infected target cell. Increased antigen expression could be achieved by enabling the vector to self‐amplify in one additional round of genome replication after cell entry (so‐called single‐cycle adenoviruses), or by using replication‐competent adenoviruses. The former approach has been explored through deletion of a structural gene (pIIIa) from a replication‐competent adenovirus^69^ that renders the virus unable to spread. The subsequent virus expresses early viral genes and replicates its genome, producing ~ 30 to 100‐fold more copies of the antigen expression cassette than replication‐deficient vectors. Impressively, a single‐cycle adenovirus encoding influenza A haemagglutinin showed a significantly higher antibody induction than its replication‐deficient equivalent, even at a 10‐fold lower dose.^70^, ^71^ One drawback of this method, however, is the requirement for a *trans*‐complementing cell line (in this case expressing pIIIa) for the production of such a single‐cycle adenovirus, which may represent a bottleneck for clinical development.

To fully exploit the advantages of a self‐amplifying vaccine, several groups have also examined the use of replication‐competent adenovirus vectors, which were administered by varying mucosal routes in permissive species (mice are not permissive for human or simian adenoviruses.) Unfortunately, results with regard to induction of humoral immunity were mixed^72^, ^73^, ^74^ or disappointing.^75^, ^76^, ^77^ One beneficial feature of replication‐deficient adenoviral vectors is the fact that the transgene is typically immuno‐dominant by default, as the lack of adenoviral gene expression precludes immune competition. It is therefore likely that replication‐competent vectors can only be effective vaccines if the transgene remains immuno‐competent while competing with numerous other viral gene products; achieving this has been challenging (reviewed in ref. 78). Furthermore, replicating adenoviral vectors carry higher safety risks because of their ability to cause systemic infection in certain susceptible populations.^79^ However, although they are indeed not suitable for use in the severely immunocompromised, a live (oral) adenovirus vaccine used to protect against respiratory disease caused by Ad4 and Ad7 has nevertheless long been used in the United States army, with a very good safety profile.^80^

In addition to exploiting vector amplification to increase antigen expression, several other methods have been described that aim to enhance the immunogenicity of adenoviral vectors. One of these approaches, antigen capsid incorporation, has been particularly successful in preclinical studies (reviewed in ref. 81). Here, antigenic epitopes are incorporated into viral structural proteins in such a way that they are exposed on the virus surface and are therefore able to elicit robust antibody responses. Epitopes from a variety of different pathogens have been tested (e.g. HIV‐1,^82^ influenza A,^83^ *Plasmodium falciparum* 84). One group, for example, demonstrated that a multivalent HIV‐1 vector based on AdHu5 elicited antibody responses to an externally presented HIV‐1 B‐cell epitope, in addition to cellular response to the virally encoded HIV‐1 gag antigen.^85^ One clear disadvantage of this method is that only short heterologous sequences can be inserted into viral structural genes without affecting virus assembly or stability. As an exception, the adenoviral capsid protein pIX can accommodate C‐terminal fusions with larger antigens. However, these fusions can have a destabilizing effect on the virion.^86^ Overall, considering the large numbers of publications reporting encouraging results, it is surprising that (to our knowledge) the capsid‐display approach has not yet been evaluated in the clinic.

## Vesicular stomatitis virus

The use of vesicular stomatitis virus (VSV) as a vaccine vector was pioneered by Rose and colleagues in the late 1990s,^87^ and the vector has since been employed in numerous preclinical studies. However, due to the challenges encountered in developing a sufficiently attenuated, safe VSV backbone, a first‐in‐human evaluation of an recombinant VSV (rVSV) vaccine did not take place until 2011.^88^ This was soon followed by clinical trials of an rVSV‐vectored Ebola vaccine (2014), which was the most advanced vaccine candidate in the recent Ebola virus epidemic in West Africa. Buoyed by this success, the VSV vector has been the subject of much interest by numerous investigators.

Vesicular stomatitis virus is an enveloped bullet‐shaped virus that belongs to the *Rhabdovirus* family and contains an 11‐kb negative‐sense RNA genome. Apart from its ability to induce robust cellular and humoral immunity against encoded transgenes, its high titre growth in validated cell lines (e.g. Vero) and the lack of a DNA intermediate during viral replication add to its attractiveness as a vaccine vector. However, owing to its negative‐sense RNA genome, rescue of recombinant virus from plasmid DNA is more challenging than rescue of DNA viruses, as it involves co‐transfection of five plasmids into a permissive cell line.^89^ The cargo capacity of rVSV vectors was found to be at least 4·5 kb,^90^ and its genomic structure conveniently allows insertion of transgenes at multiple sites, which will result in transgene expression at varying levels. Unlike adenoviral vectors (which are typically replication‐deficient), most VSV vaccine vectors are replication‐competent, albeit attenuated. Attenuation represents an important safety feature, as wild‐type VSV is neurovirulent upon intracranial inoculation.^91^ Attenuation of VSV can be achieved in several ways; the most prominent example combines down‐regulation of N protein expression with truncation of the VSV‐G cytoplasmic tail, resulting in the attenuated vector rVSVN4CT1, which has been approved for clinical studies.^92^

In the past 20 years, VSV vaccine vectors have been demonstrated to induce robust cellular and humoral immune responses in numerous preclinical studies, leading to protection in many animal models of pathogen challenge, frequently after a single vector administration (reviewed in ref. 93). Durability of a protective immune response (up to 1 year, so far) has also been demonstrated.^94^ Since 2011, rVSV vectors have been evaluated in four completed or ongoing clinical trials for HIV‐1,^88^, ^95^, ^96^, ^97^ and in Phase I, II and III trials for Ebola (reviewed in ref. 98). Of note, the Phase III trial that took place during the 2014/15 outbreak in Guinea showed promising efficacy in a ring‐vaccination strategy.^99^ Analysis of immunogenicity in these studies revealed a robust induction of neutralizing antibodies and a modest CD8^+^ response for all participants in the Ebola virus clinical trials,^100^ whereas the only report of an HIV‐1 trial was more disappointing, with modest CD4^+^ levels in two‐thirds of participants and low antigen‐specific antibody levels in one‐third of participants.^88^ Significant differences existed in the vector backbones used in these trials: rVSV‐HIV‐1 vectors were attenuated by genetic engineering as mentioned above (containing the rVSVN4CT1 backbone) and the HIV‐1 antigen coding sequence was placed in position 1 of the genome. In contrast, the rVSV‐EBOV clinical vector was simply based on the cell culture adapted VSV Indiana strain lacking VSV‐G (rVSVΔG), with the Ebola glycoprotein (EBOV‐GP) placed in position 4. In the absence of the native glycoprotein, EBOV‐GP acted as the vaccine antigen in addition to the viral entry protein. As the VSV glycoprotein is the main determinant of viral tropism, safety considerations for the rVSV‐EBOV vector are different from those for the rVSV‐HIV vectors. In initial Phase I trials, replication of the rVSV‐EBOV vector was detected in synovial fluid and skin lesions, most probably due to EBOV‐GP‐specific tissue tropism.^101^

In an effort to address safety concerns, more attenuated, second‐generation rVSV‐EBOV vectors were recently developed.^102^ Attenuation was achieved by employing the rVSVN4CT1 backbone, and reduced virus growth was demonstrated in cell culture.^102^ The vaccine was still protective in a non‐human primate Ebola virus challenge model after a single administration and, reassuringly, vaccination resulted in a 10‐fold to 50‐fold reduction in vaccine‐associated viraemia in the blood compared with first‐generation vectors in previous studies. Unfortunately, this study did not include a direct comparison with first‐generation rVSV‐ZEBOV. However, a Phase I trial of this vaccine (rVSVN4CT1‐EBOVGP1) was recently completed,^103^ and so insight into the safety profile of this vector will soon be available. In addition, GemEvac‐Combi, an Ebola virus vaccine containing an rVSV expressing the Ebola glycoprotein, has also been developed.^104^ This vaccine was evaluated for safety and immunogenicity in a 2015 Phase I trial and subsequently licensed by the Ministry of Health of the Russian Federation. However, it is difficult to assess the potential impact of this vaccine, considering the paucity of published preclinical data and the lack of information regarding vector construction. A Phase 4 study of this vaccine involving 2000 volunteers in Russia and Guinea is planned for 2017–2019.^105^

In parallel with ongoing clinical studies, VSV vectors have been modified to further improve utility to create multivalent vectors. Mire *et al*. generated trivalent rVSV encoding glycoproteins from Zaire Ebola virus, Sudan Ebola virus and Marburg virus, and demonstrated protection against all three virus strains in a guinea pig challenge model after a single immunization, even though antibody responses to each antigen differed in magnitude.^106^ Encouragingly, vectors containing ~ 6 kb transgenic cargo were generated, suggesting that multiple or large inserts can be incorporated into rVSV vectors. Another recent valuable observation regarding rVSV vector development is their ability to provide protection even after exposure to the pathogen. For example, full protection was shown when rhesus monkeys were vaccinated with an rVSV‐MARV vector 30 min after receiving a lethal dose of Marburg virus,^107^ and five of six animals were still protected when vaccinated 24 hr after challenge.^108^ T‐cell depletion had no impact on vaccine‐induced protection,^109^ suggesting that rapid induction of antibodies may underlie vaccine efficacy. Taken together, both preclinical and clinical studies suggest that the strengths of the VSV vector lie in its attenuated replicative capacity and its ability to elicit high and durable antibody levels to surface‐displayed antigens; characteristics that make it a promising vaccine vector for emerging or outbreak‐prone viral diseases.

## Measles virus

Despite its pathogenicity, the development of measles virus (MV) as a vaccine vector was initiated in the late 1990s. This was based on the large success of the live attenuated measles vaccine itself. Measles vaccines were developed in the early 1960s by cell culture adaptation of wild‐type virus isolates, leading to attenuation through an accumulation of mutations. The most attenuated strains, still used today, have excellent safety profiles while still inducing extremely durable, protective antibody‐ and T‐cell‐mediated immunity in 95% of recipients after a single vaccination.^110^, ^111^, ^112^ Interestingly, T‐cell‐mediated responses to MV are predominantly of the CD4^+^ phenotype,^113^ unlike the CD8^+^ dominated response to adenoviral vectors,^114^ which may have important implications when considering these vector platforms for vaccine development.

A member of the *Paramyxovirus* family, MV is an enveloped spherical virus and contains a 16‐kb negative‐sense RNA genome. The development of reverse genetics tools and rescue of MV from cDNA in 1995^115^ accelerated both basic MV virology research and exploration of MV as a vaccine vector. Studies demonstrated a cargo capacity in excess of 6 kb and an excellent induction of humoral and cellular immune responses against encoded transgenes.^116^ In addition, MV was easily adaptable to large‐scale bio‐manufacture at low production cost. Recombinant MVs expressing one or more genes from heterologous pathogens have now been used in numerous preclinical vaccine studies (reviewed in ref. 117). One group, for example, generated an rMV expressing HIV‐1 Gag, RT and Nef as a fusion protein (MV1‐F4) and assessed immunogenicity of the vector in prime or prime‐boost regimens in cynomolgus macaques.^118^ Vaccination induced robust antigen‐specific CD4^+^ and modest CD8^+^ T‐cell responses, and high levels of antibody reactive to exogenous antigens and MV‐encoded proteins that were further amplified after boosting.^118^

During preclinical development, concerns arose regarding the impact of pre‐existing immunity against the MV vector, as the live attenuated measles vaccine is part of routine childhood immunization programmes in many countries. However, higher doses or alternative administration routes of the vector can overcome existing anti‐measles antibody levels in mice.^119^ Of note, in this study pre‐existing immunity was artificially modelled using intravenous administration of anti‐measles antibodies. However, another study that examined previous exposure to attenuated measles vaccine found no influence of existing anti‐MV immunity on transgene immunogenicity in mice or macaques.^120^ Another important consideration for the possible paediatric use of MV vectors is the requirement for the vector to retain vaccine competence against MV itself. This was demonstrated in a macaque model using an MV‐based hepatitis B vaccine candidate.^121^

After almost two decades of preclinical development, MV vaccine vectors have recently been advanced into clinical trials, with two Phase I studies completed (HIV‐1,^122^ Chikungunya virus (CHIKV)^123^) and two Phase II CHIKV trials ongoing or planned in Europe and Puerto Rico, respectively.^124^, ^125^ In the Phase I CHIKV study, volunteers received escalating priming doses followed by a booster dose of an rMV encoding the structural genes (C, E3, E2, 6K and E1) of CHIKV, a mosquito‐borne alphavirus of the tropics and sub‐tropics that is threatening to become a global public health burden. Protective immunity against CHIKV is antibody‐mediated in a mouse model.^126^ Encouragingly, seroconversion was demonstrated for 90% of participants in the high‐dose group after one immunization, and for all participants after the second vaccination. In addition, immunogenicity was not affected by pre‐existing anti‐measles immunity, an important finding that will hopefully be confirmed in larger ongoing studies.

## Conclusions and future perspective

**Figure 1:**
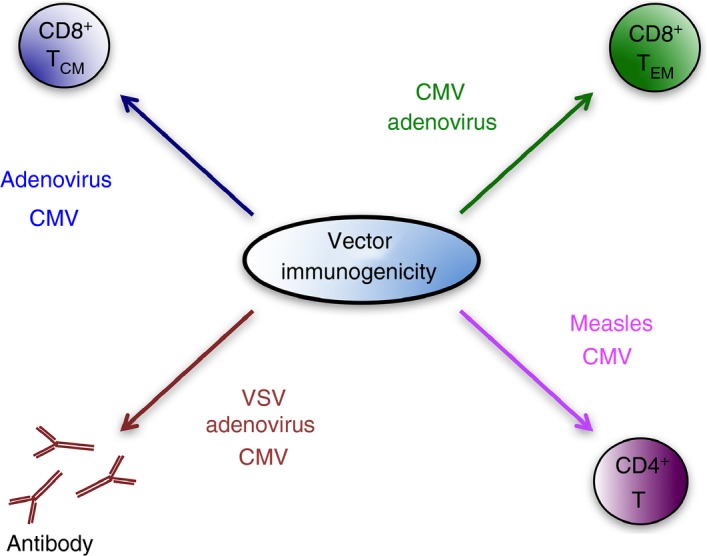
Viral vector‐induced immune responses. Schematic of relative induction of adaptive immune responses by different viral‐based vaccine vectors. Text size represents relative induction of adaptive immunity. CMV, cytomegalovirus; VSV, vesicular stomatitis virus.

Viral vectors hold much promise for vaccine vector development to counter infectious diseases. Significant advances have been made regarding the production of immunogenic vectors that can be used in individuals with previous immunity to the viruses on which vectors are based. More detailed understanding of which immune responses are preferentially induced by vectors and how they are triggered will inform decisions as to which vectors are most relevant for vaccination against a specific infectious disease (Fig. 1). Safety considerations remain a significant challenge in the development of certain viral vectors. This is relevant not only for vaccination against infectious diseases, but also for the potential exploitation of virus‐based vectors in cancer vaccination strategies where individuals are often immune compromised. Hence, understanding better how to balance safety and immunogenicity will have broad implications for the management of infectious diseases and beyond.

## Disclosures

The authors declare no competing interests.
